# Nanoparticle-Based Antimicrobials: Surface Functionality is Critical

**DOI:** 10.12688/f1000research.7595.1

**Published:** 2016-03-16

**Authors:** Akash Gupta, Ryan F. Landis, Vincent M. Rotello

**Affiliations:** 1 Department of Chemistry, University of Massachusetts Amherst, 710 North Pleasant Street, Amherst, Massachusetts, 01003, USA

**Keywords:** Antimicrobials, nanoparticles, bacteria, biofilms, antibacterial

## Abstract

Bacterial infections cause 300 million cases of severe illness each year worldwide. Rapidly accelerating drug resistance further exacerbates this threat to human health. While dispersed (planktonic) bacteria represent a therapeutic challenge, bacterial biofilms present major hurdles for both diagnosis and treatment. Nanoparticles have emerged recently as tools for fighting drug-resistant planktonic bacteria and biofilms. In this review, we present the use of nanoparticles as active antimicrobial agents and drug delivery vehicles for antibacterial therapeutics. We further focus on how surface functionality of nanomaterials can be used to target both planktonic bacteria and biofilms.

## Introduction

Bacterial infections cause 300 million cases of severe illness every year with 16 million, including 2 million children, killed ^1^. Infections caused by multi-drug-resistant (MDR) bacteria greatly increase the threat generated by bacterial infections. In addition to acute illness, bacterial infections can result in chronic disease states, where bacterial colonization develops into a biofilm, a complex three-dimensional bacterial community ^2^. The complexity of the biofilm matrix makes biofilm-associated diseases more clinically challenging than planktonic bacteria in both diagnosis and treatment ^3^. Finally, there has been a significant decrease in the number of approved antibiotics recently, contributing to the urgency of developing alternative antimicrobial agents ^4^.

**Figure 1.**
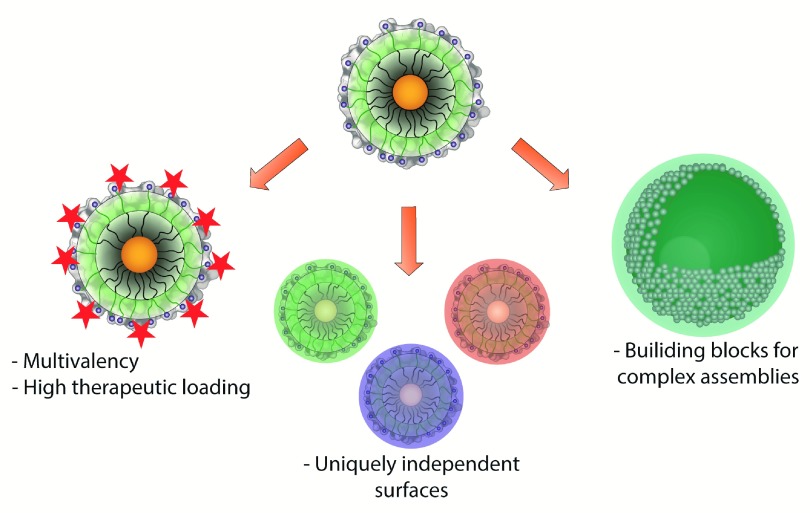
Nanoparticles as scaffolds and building blocks for antimicrobial agents.

Nanoparticles (NPs) are emerging as weapons in our antimicrobial arsenal owing to their unique nanoscale physical and chemical properties ^5, 6^. For example, NP size is commensurate with biomolecular and bacterial cellular systems, providing a platform where nanomaterial-bacteria interactions can be fine-tuned through appropriate surface functionalization ^7, 8^. Moreover, the high surface area to volume ratio of nanomaterials enables high loading of therapeutics, with promising synergy arising from multivalent interactions. NPs provide a way to address the common mechanisms of antibiotic resistance, such as permeability regulation ^9, 10^, multi-drug efflux pumps ^11^, antibiotic degradation ^12, 13^, and target site binding affinity mutations ^14^. NPs also provide alternative pathways to combat biofilm/MDR infections and significantly lower bacteria resistance over time ^15– 17^. NPs utilize multiple mechanisms to kill bacteria, making it difficult for them to adapt existing strategies for developing resistance ^18^. Following this strategy, several NP-based systems have been developed to improve antimicrobial efficacy ( Figure 1) ^19– 21^. In this review, we will focus on recent studies that use engineered NPs as active therapeutic agents or as delivery vehicles to transport drugs to the site of infection.

## Nanoparticle interactions with bacteria and biofilms

Engineering the interactions of nanomaterials with bacteria/biofilm matrices plays a crucial role in designing NP-based antimicrobial systems. The surface properties of NPs are highly versatile and can be easily modulated through ligand engineering to generate particles with new and emergent properties ^22– 24^. These NPs can be utilized for not only therapeutic applications but also fundamental studies on bacterial behavior. In early studies of NP-microbe interactions, Rotello and co-workers showed that cationic gold NPs (AuNPs) possessed toxicity against bacteria ^25^. Subsequently, they demonstrated that hydrophobic, cationic AuNPs developed spatiotemporal aggregate patterns on the bacterial surface. The aggregate patterns depended upon the nature of the bacteria as well as the size of the NPs. In this work, 6 nm AuNPs were found to have low toxicity, whereas 2 nm AuNPs rapidly lysed *Bacillus subtilis* but not *Escherichia coli* ^26^. In a similar study, Feng and co-workers further corroborated the fact that NP and bacterial surface chemistry impact NP-bacteria interactions and toxicity. They reported that the NPs with maximal cationic charge associated most significantly with the bacterial surfaces, inducing the greatest membrane damage and toxicity ^27^. These studies provide valuable insight into designing therapeutic constructs for planktonic bacteria treatment.

Bacteria can self-colonize to form biofilms. Biofilm infections are difficult to treat because the extracellular matrix produced by bacteria creates a microenvironment within the host. This allows bacteria to evade immune responses and dramatically increase resistance to traditional antibiotic treatments ^28, 29^. The complex architecture, dynamics, and composition of extracellular polymeric substances (EPS) in the matrix are profoundly responsible for the low penetration of therapeutic agents ^30^. Diffusion of therapeutics inside the biofilm can be affected by several genetic and physiological heterogeneities such as the hydrophobicity of bacterial cell walls ^31^. Hence, fundamentally understanding the interactions between NPs and complex biofilm matrices is crucial in designing materials for biofilm treatment.

**Figure 2.**
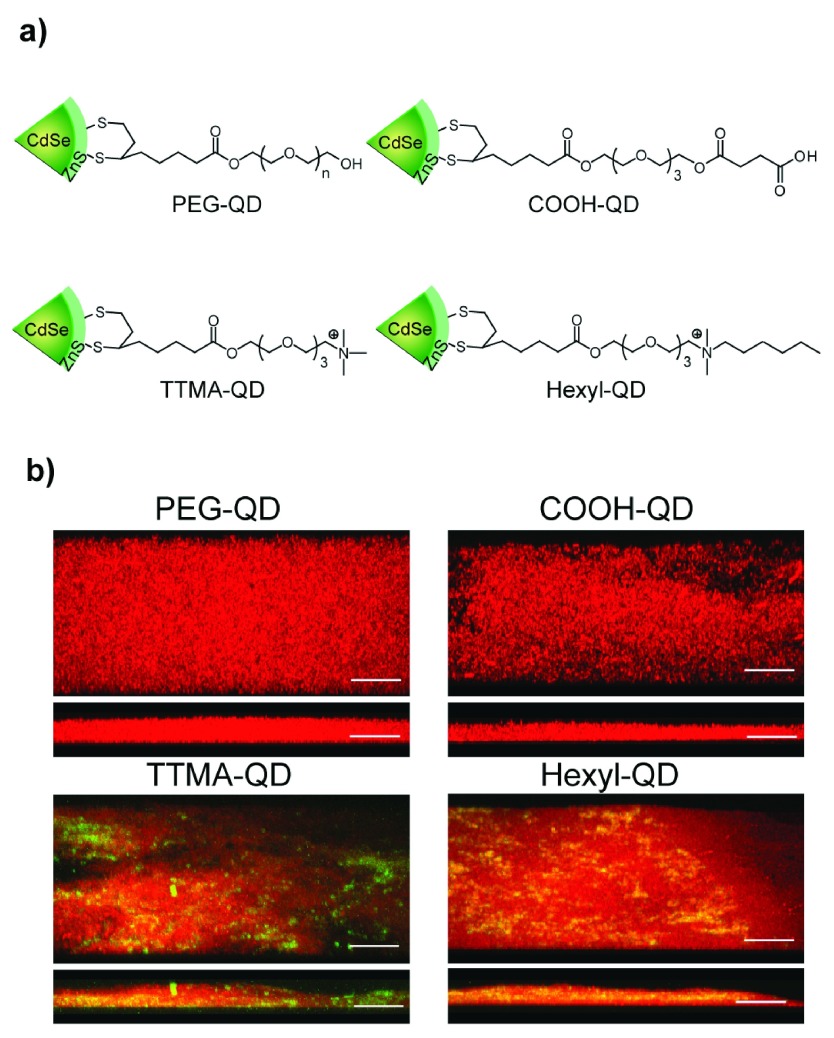
Surface design controls penetration ability of nanoparticles. **a**) Quantum dots used in study. **b**) Micrographs of microtomed slices of the biofilm showing no penetration by anionic and neutral particles and efficient infiltration by cationic quantum dots ^33^.

The penetration and deposition of NPs within the biofilms are key components for the design of biofilm therapeutics. Peulen and Wilkinson reported that the penetration ability of NPs decreased inversely to their size due to small pore sizes within biofilms ^32^. Furthermore, NP deposition inside the biofilms is largely dependent upon the electrostatic interaction as well as the homogeneity of the charges across the biofilm surface. In a related study, Rotello and co-workers provided further insight on the penetration ability of the NPs inside the biofilms. They demonstrated that the neutral and anionic quantum dots (QDs) did not show any penetration inside the biofilms, while cationic QDs were widely distributed throughout the biofilm. Furthermore, cationic QDs with hydrophobic terminal groups were found inside the bacterial cells, whereas their hydrophilic counterparts remained in the EPS matrix of the biofilm ( Figure 2) ^33^.

## Nanoparticles as active antimicrobial agents

NPs provide multiple attributes that facilitate the development of unique antimicrobial strategies ^34, 35^. NPs can interact with and penetrate bacterial cells with unique bacteriostatic and bactericidal mechanisms ^36^. For example, possessing slightly larger diameters than drug efflux pumps, NPs can potentially reduce efflux-mediated extrusion ^37, 38^. Exploiting these characteristic properties, several NP-based systems have been employed for antimicrobial applications. Xu and co-workers demonstrated enhanced *in vitro* antibacterial activities of vancomycin-capped AuNPs (Au-Van) against vancomycin-resistant *enterococci* and *E. coli* strains ^39^. Similarly, Feldheim and co-workers demonstrated that antimicrobial activity of NPs functionalized with non-antibiotic molecules depended upon their composition on the surface ^40^. These studies indicate that modulating NP surfaces exhibits great potential for antimicrobial therapy. However, further studies on how NP surface functionality modulates antimicrobial activity can provide valuable information for future NP-based antimicrobial agents.

**Figure 3.**
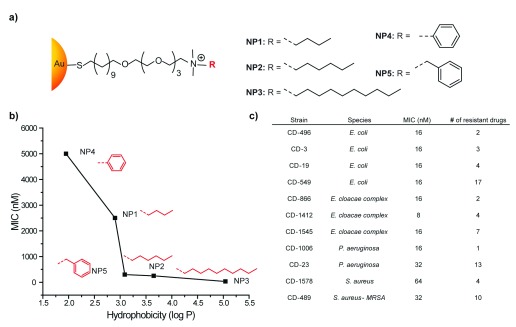
Functionalized gold nanoparticles as antimicrobial agents. **a**) Nanoparticles studied, featuring 2 nm gold cores. **b**) Toxicity of nanoparticles to a laboratory *Escherichia coli* strain. **c**) Minimum inhibitory concentrations of nanoparticle 3 against multi-drug-resistant bacteria ^41^.

In a recent study, the Rotello group reported a strategy to combat MDR bacteria by engineering the ligands on NP surfaces. Cationic and hydrophobic functionalized AuNPs effectively suppressed the growth of 11 clinical MDR isolates at low concentrations ( Figure 3). The minimum inhibitory concentrations (MICs) observed for these systems with most bacteria strains was 16 nM. Moreover, bacteria strains did not develop resistance against NPs, even after 20 passages at sub-MIC concentrations, which is far beyond that of traditional antibiotics ^41^. Overall, this study provides an excellent starting platform to design antibacterial therapeutics in future studies.

The antibacterial activity of silver has been well established. High surface area and concomitant increase in dissolution rate are key to its use in silver-based antimicrobials, where free Ag ^+^ ions are the active agents ^42^. However, they face certain shortcomings, such as high toxicity to mammalian cells and limited penetration in biofilm matrices ^43, 44^. Recent studies have focused on countering these issues by using inherent NP properties and surface functionalization as their toolkit. For example, Mahmoudi and co-workers developed silver ring-coated superparamagnetic iron oxide NPs (SPIONS) with ligand gaps that demonstrated high antimicrobial activity and remarkable compatibility with healthy cells. Additionally, these NPs exhibited enhanced activity against biofilm infections due to deeper penetration under an external magnetic field ^45^.

**Figure 4.**
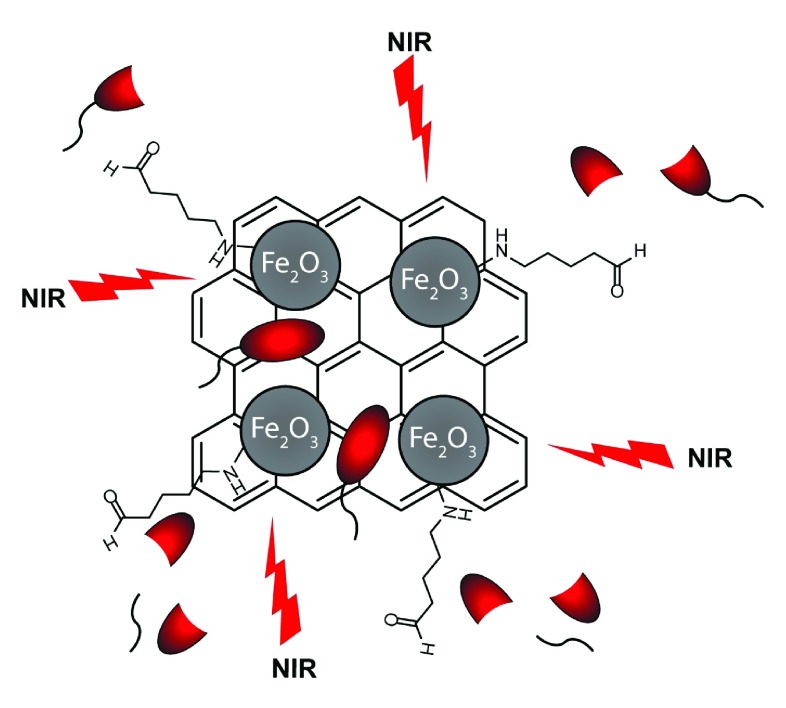
Schematic representation of antibacterial photothermal treatment by mildly reduced graphene oxide functionalized with glutaraldehyde.

Graphene NPs ^46^, AuNPs ^47^, and carbon nanotubes ^48^ possess photothermal properties that can be utilized to design therapeutic agents. These nanomaterials absorb light (700–1100 nm) and release heat. Ling and co-workers designed graphene-based photothermal NPs that captured and killed *Staphylococcus aureus* and *E. coli* bacteria upon near-infrared (NIR) laser irradiation. In this approach, graphene oxide was reduced and functionalized with magnetic NPs (MRGO). These NPs were functionalized with glutaraldehyde (GA) to induce excellent crosslinking properties with Gram-positive and Gram-negative bacteria ( Figure 4). Rapid and effective killing of 99% of both bacterial species was achieved upon NIR irradiation ^49^.

## Nanoparticles as drug delivery vehicles for antibacterial therapy

Bacterial infections are able to evade antibiotic treatment through reduced bactericidal concentration or reduced antimicrobial activity of therapeutic agents at the site of infection ^50, 51^. Localized delivery of the drugs/antimicrobials can increase their therapeutic efficacy. Therefore, NPs can serve as promising drug delivery vehicles owing to their tunable surface functionality, biocompatibility, and high drug loading capacity ^17^.

NPs such as mesoporous silica possess a uniquely large surface area and tunable pore size that make them promising candidates for designing drug delivery vehicles ^52^. For example, Schoenfisch and co-workers designed amine-functionalized silica NPs that were able to readily penetrate and eradicate pathogenic biofilms through rapid nitric oxide release ^53^. Similarly, silica NPs have been fabricated as scaffolds for silver NP (AgNP) release ^54^. Using NPs for controlled antimicrobial release can markedly improve their biocompatibility with mammalian cells and mitigate their hazardous environmental impact ^55– 57^. In one such study, biodegradable lignin-core NPs (EbNPs) infused with silver ions were proposed as greener alternatives to AgNPs. EbNPs were coated with cationic polyelectrolytes and loaded with Ag ^+^ ions. These NPs exhibited broad-spectrum biocidal action against Gram-positive and Gram-negative bacteria at lower Ag ^+^ ion concentrations than conventional AgNPs ^58^.

Therapeutic selectivity is critical when designing effective drug delivery vehicles. Triggered release of antimicrobials from these nanocarriers can be an alternative strategy to diminish their undesirable side effects ^59, 60^. In one particular study, Langer and co-workers designed PLGA-PLH-PEG NPs as a carrier to deliver vancomycin to bacterial cells, exploiting their localized acidity. PLGA-PLH-PEG NPs demonstrated high binding affinity to bacterial cells at pH 6.0 as compared to 7.4. Vancomycin-encapsulated NPs exhibited a 1.3-fold increase in the MIC against *S. aureus* as compared to 2.0-fold and 2.3-fold for free and PLGA-PEG-encapsulated vancomycin, respectively ^61^. In a similar study, pH-responsive NPs were used to deliver hydrophobic drugs to biofilm moieties. Polymeric NPs used in this study consisted of a cationic outer shell to bind with the EPS matrix and a pH-responsive hydrophobic inner shell to release encapsulated farnesol molecules on demand. These scaffolds resulted in a 2-fold increase in efficacy in the treatment of biofilms as compared to the drug alone ^62^.

Apart from acidic microenvironments, NPs can be designed to trigger antibiotic release upon exposure to bacterial toxins. For example, Zhang and co-workers designed AuNP-stabilized phospholipid liposomes (AuChi-liposomes) that respond to bacterial toxins. Chitosan-functionalized AuNPs were adsorbed on the liposomal surfaces to provide stability and prevent undesirable antibiotic leakage. In the presence of α-toxin-secreting *S. aureus* bacteria, AuChi-liposomes released vancomycin that effectively inhibited their growth ^63^.

**Figure 5.**
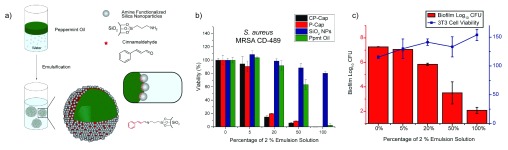
Nanoparticle-stabilized capsules for treatment of biofilm infections. **a**) Fabrication of capsules. **b**) Efficacy of cinnamaldehyde dissolved in peppermint oil capsule (CP-Cap) and controls against a clinical isolate of methicillin-resistant *Staphylococcus aureus*. **c**) Toxicity of CP-Cap against *Escherichia coli* cells while enhancing fibroblast viability ^66^.

Cationic NPs exhibit excellent penetration ability in biofilms ^64^. Moreover, they can self-assemble at the oil-water interfaces to generate nanocapsules ^65^. Combining these two characteristic features, Rotello and co-workers generated a highly effective therapeutic system for the treatment of bacterial biofilm infections. Peppermint oil and cinnamaldehyde were chosen as the therapeutic oil template, owing to their inherent antimicrobial nature, in combination with amine-functionalized cationic silica NPs that stabilized the oil-water interface to generate nanocapsules (CP-caps) ( Figure 5). These capsules were further stabilized by the formation of hydrophobic Schiff bases upon reacting with cinnamaldehyde. The cationic NPs enabled the capsules to readily penetrate the biofilms and release the antimicrobial oils to eradicate the biofilm infections. Moreover, the therapeutic selectivity of CP-caps was tested on a biofilm-fibroblast cell co-culture model. These studies showed effective biofilm infection eradication with simultaneous growth enhancement of fibroblast cells ^66^.

High therapeutic selectivity makes these capsules useful antimicrobial agents for topical administration. Use of these nanomaterials systemically, however, requires an understanding of NP pharmacokinetics (PK) and biodistribution (BD). The PK and BD properties of NPs depend on several factors such as their size, shape, and surface functionalization ^67, 68^. Apart from their physiochemical characteristics, the administration route of NPs likewise determines their systemic or local effect. For example, intravenous injection is used for targeting the liver and spleen, whereas mucoadhesive NPs are used for oral and nasal drug delivery ^69^. Similarly, uptake and elimination of NPs in cells/tissues are dependent upon their physiochemical properties ^70^. For example, cationic NPs have higher uptake and slower rate of exocytosis in cells as compared to their anionic counterparts ^25, 71^. Hence, evaluating the PK behavior of the current antimicrobial systems is important for their translation into the clinic.

## Conclusion

NPs provide a versatile platform in designing materials for antimicrobial therapy. Tunable surface functionality and multivalency makes them promising candidates to target planktonic bacteria. Moreover, excellent biofilm penetration enhances their activity towards a range of biofilm-based infections. NP-based antimicrobial agents can be readily used for *ex vivo* applications such as sterilizers for surfaces and devices. The most accessible target in the near future includes the topical applications of NP-based systems for wound healing. However, further studies at the fundamental, biological, and pharmacological level are required to enable systemic administration of these antimicrobials. In conclusion, NPs have offered promising avenues to design effective next-generation therapeutics against bacterial threats.

## Abbreviations

AgNP, silver nanoparticle; AuChi-liposomes, AuNP stabilized phospholipid liposomes; AuNPs, gold nanoparticles; Au-Van, vancomycin-capped AuNPs; CP-caps, cinnamaldehyde dissolved in peppermint oil capsules; EbNPs, lignin-core nanoparticles; EPS, extracellular polymeric substances; GA, glutaraldehyde; MDR, multi-drug resistance; MIC, minimum inhibitory concentration; MRGO, mildly reduced graphene oxide functionalized with magnetic nanoparticles; NIR, near-infrared; NPs, nanoparticles; PK, pharmacokinetics; PLGA-PLH-PEG, poly(D,L-lactic-co-glycolic acid)-b-poly(L-histidine)-b-poly-(ethylene glycol); QDs, quantum dots; SPIONS, superparamagnetic iron oxide nanoparticles.
